# Nanozymes for periodontal biofilm-associated challenges: mechanisms, platforms, and translational perspectives

**DOI:** 10.3389/fbioe.2026.1965283

**Published:** 2026-09-21

**Authors:** Yujie Ji, Tianyu Huang, Qiudong Xu, Fangyong Zhu, Zelda Ziyi Zhao

**Affiliations:** 1 Wuxi Stomatology Hospital, Wuxi, China; 2 Department of Stomatology, Nantong First People’s Hospital, Nantong, China; 3 Department of Stomatology, Affiliated Hospital of Jiangnan University, Wuxi, China

**Keywords:** antimicrobial therapy, biofilm, EPS degradation, nanozymes, periodontitis, reactive oxygen species

## Abstract

Periodontitis is a chronic inflammatory disease driven by dysbiotic subgingival biofilms whose extracellular polymeric substance (EPS) matrix confers marked resistance to conventional therapies and host immunity, with antibiotic resistance further compounding the challenge. Nanozymes, as nanomaterials with intrinsic enzyme-like catalytic activities, offer a promising alternative due to their catalytic efficiency, stability, and multifunctionality. This narrative review synthesizes current evidence on nanozyme applications for overcoming biofilm-associated challenges in periodontitis, focusing on antibiofilm mechanisms, representative platforms, delivery strategies, and translational hurdles. A literature search was conducted across PubMed, Web of Science, and Scopus using keywords including “nanozyme,” “periodontitis,” and “biofilm.” Nanozymes combat periodontal biofilms through complementary mechanisms: reactive oxygen species (ROS) generation via peroxidase- and oxidase-like activities to kill biofilm-embedded bacteria; degradation of EPS components including polysaccharides, proteins, and extracellular DNA; disruption of bacterial adhesion and quorum sensing; and modulation of inflammation via ROS scavenging through catalase- and superoxide dismutase-like activities. Representative platforms have been evaluated in diverse preclinical models including marginal periodontitis, apical periodontitis, and other biofilm-associated infections each with distinct levels of direct periodontal relevance. While some platforms have been validated directly in periodontitis models, others provide indirect evidence or mechanistic insights that inform translational potential. These include Ru-C_3_N_4_ single-atom nanozymes (tested in otitis media models), AgAu-CeO_2_ heterojunction nanozymes with probiotics, ferumoxytol (tested in apical periodontitis), mitochondria-targeted ferritin nanozymes, and Ru@COF nanozymes. Despite encouraging preclinical evidence, challenges in long-term biosafety, metal-ion accumulation, and clinical translation persist. Future research should prioritize standardized safety evaluation, biodegradable designs, and clinical validation to translate nanozyme-based therapies from bench to bedside.

## 1 Introduction

Periodontitis is a chronic inflammatory disease affecting approximately 62% of dentate adults worldwide, with over one billion people suffering from severe forms of the disease (Perez et al., 2024; Kassebaum et al., 2014). The disease is fundamentally driven by a dysregulated host immune response to pathogens within subgingival biofilms, leading to the destruction of periodontal ligament collagen, resorption of alveolar bone, and ultimately tooth loss (Hajishengallis, 2015; Kinane et al., 2017). The formation of pathogenic plaque biofilms is a key driver of disease progression, and the extracellular polymeric substance (EPS) matrix provides structural support and a protective barrier that renders these biofilms markedly resistant to conventional antimicrobial strategies (Flemming and Wingender, 2010; Zhao et al., 2023).

Within dental plaque biofilms, bacteria are encapsulated by the EPS, forming a dense physical barrier that impedes drug penetration and maintains bacteria in a low-metabolic state, significantly reducing their susceptibility to conventional antimicrobial agents (Stewart, 2002; Fulaz et al., 2019). Furthermore, long-term or repeated use of antibiotics during treatment may lead to bacterial resistance and disruption of the oral microbiota. These limitations underscore the urgent need for novel therapeutic strategies that can effectively disrupt biofilms, overcome resistance mechanisms, and promote tissue regeneration (Mishra et al., 2023; Asare et al., 2022).

Nanozymes, nanomaterials with intrinsic enzyme-like catalytic activities, have attracted increasing attention as promising tools for combating biofilm-related infections (Gao et al., 2007; Wei and Wang, 2013). Since the first report of Fe_3_O_4_-based nanozymes in 2007, over 1,200 types of nanomaterials have been identified to exhibit enzyme-mimicking activities (Jeyachandran and Lopes, 2023; Zhou and Wang, 2022; Wei and Wang, 2013). As artificial enzyme mimics, nanozymes exhibit excellent catalytic efficiency, enabling them to rapidly generate reactive oxygen species (ROS) or catalyze the hydrolysis of biomolecules within microbial cells and biofilms, thereby effectively preventing biofilm formation or eradicating established biofilms (Chen et al., 2018; Gao et al., 2021). Nanozymes offer several distinct advantages over conventional antimicrobials, including high stability against temperature and proteolytic degradation, tunable catalytic activities through surface engineering, the ability to integrate multiple enzyme-like activities on a single particle, and antimicrobial mechanisms primarily dependent on ROS-mediated oxidative damage rather than specific molecular targets, making them less prone to resistance.

Nanozymes can be broadly classified based on their catalytic activities into several categories: peroxidase-like nanozymes (e.g., Fe_3_O_4_, CeO_2_, Prussian blue) that catalyze H_2_O_2_ decomposition to generate OH; oxidase-like nanozymes that reduce O_2_ to superoxide; catalase-like nanozymes that decompose H_2_O_2_ to H_2_O and O_2_; and SOD-like nanozymes that dismutate superoxide to H_2_O_2_ (Wei and Wang, 2013; Jeyachandran and Lopes, 2023). Additionally, some nanozymes exhibit hydrolase-like activities, including DNase and protease mimicry, enabling direct degradation of biofilm matrix components (Li et al., 2022; Pan et al., 2022). Based on their catalytic actions, nanozymes can be broadly grouped into oxidoreductase-mimetic types (including peroxidase-, oxidase-, catalase-, and SOD-like activities) that primarily generate or scavenge reactive oxygen species, and hydrolase-mimetic types that directly degrade biofilm matrix components such as polysaccharides, proteins, and extracellular DNA. The ability to integrate multiple catalytic activities on a single nanoplatform is a hallmark advantage of nanozyme design, enabling simultaneous antibacterial, antibiofilm, antioxidant, and tissue-regenerative functions particularly suited to the multifaceted pathophysiology of periodontitis (Hosseini Hooshiar et al., 2024; Luo et al., 2025).

This narrative review aims to comprehensively synthesize current evidence on the application of nanozymes for overcoming biofilm-associated challenges in periodontitis, covering their antibiofilm mechanisms, representative nanozyme platforms validated in preclinical models, advanced delivery strategies, and the challenges that must be addressed for clinical translation. Of note, the evidence included in this review derives from multiple sources. Studies were classified according to their directness of periodontal relevance: (1) directly evaluated in marginal periodontitis models; (2) evaluated in apical periodontitis or other oral diseases; (3) evaluated in non-oral biofilm models (e.g., wounds, pneumonia); and (4) proposed for periodontal application based on mechanistic relevance. This classification is explicitly indicated throughout the text to provide transparency regarding the strength and source of the evidence presented.

This narrative review was conducted through a comprehensive literature search across PubMed, Web of Science, and Scopus. The search was performed using the following search string: (nanozyme OR nanozymes) AND (periodontitis OR periodontal OR periodontal disease OR biofilm). Additional targeted searches were conducted for specific platforms using terms such as “Ru-C_3_N_4_,” “AgAu-CeO_2_,” “ferumoxytol,” and “Ru@COF.” The search period covered publications from database inception to July 15, 2026. Language was restricted to English. Included publication types comprised original research articles, preclinical studies (*in vitro* and animal models), clinical studies, and systematic or narrative reviews. Exclusion criteria were: (1) studies not related to nanozymes or biofilm-associated infections; (2) studies on non-oral biofilms without clear translational relevance to periodontal applications; (3) non-English publications; and (4) editorials and commentaries without original data. Conference abstracts and supplement publications were included only when they provided substantive data that could not be found in full-length publications; such cases are explicitly noted in the text and table as evidence from conference proceedings. Titles and abstracts were screened for relevance, and full texts of potentially eligible articles were retrieved for detailed evaluation. Duplicate records were removed using reference management software (EndNote). A total of 371 records were identified, of which 86 were included in the final synthesis. As this is a narrative review, no formal quantitative quality assessment or meta-analysis was performed; instead, evidence was critically evaluated and narratively synthesized.

## 2 Periodontal biofilms: structure, pathogenicity, and therapeutic challenges

### 2.1 Biofilm formation and structure

Periodontal biofilms are complex, multispecies microbial communities that form on tooth surfaces within the subgingival environment (Kuramitsu et al., 2007; Valm, 2019). The formation of dental plaque biofilms involves sequential stages, including initial bacterial adhesion to the pellicle-coated tooth surface, microcolony formation, biofilm maturation, and eventual dispersal (Benoit et al., 2019; Zhao et al., 2023). Early colonizers such as *Streptococcus* and *Actinomyces* species create a foundation for the subsequent colonization of bridging organisms like *Fusobacterium nucleatum*, which in turn facilitate the adherence of late colonizers including *Porphyromonas gingivalis*, *Tannerella forsythia*, and *Treponema denticola*, the so-called red complex which is strongly associated with periodontitis severity (Kolenbrander et al., 2010; Valm, 2019).

The EPS matrix is the defining structural feature of mature biofilms. Composed of polysaccharides, proteins, and extracellular DNA (eDNA), the EPS provides mechanical stability, facilitates intercellular adhesion, and functions as a diffusion barrier that limits the penetration of antibiotics and host immune effectors (Flemming and Wingender, 2010; Fulaz et al., 2019). The EPS matrix not only provides structural support but also creates a protective microenvironment that shields bacteria from antimicrobial agents and host defenses, while sequestering enzymes, nutrients, and virulence factors essential for bacterial survival (Stewart, 2002; Pinto et al., 2020). Importantly, the EPS composition varies among periodontal pathogens: *P. gingivalis* produces capsular polysaccharides and gingipains that degrade host tissues, while *Streptococcus mutans* synthesizes glucan polymers via glucosyltransferases that form the scaffold of cariogenic biofilms (Lin et al., 2022; Kolenbrander et al., 2010). This compositional diversity necessitates nanozyme platforms capable of targeting multiple EPS components simultaneously, underscoring the importance of multi-enzyme-mimetic designs.

It is important to distinguish subgingival periodontal biofilms from other oral biofilm types. Periodontitis-associated biofilms are predominantly composed of Gram-negative anaerobes, particularly the red complex organisms along with other periodontal pathogens such as *Aggregatibacter actinomycetemcomitans* and *Prevotella intermedia*. These polymicrobial communities reside in subgingival pockets and drive a chronic inflammatory response that leads to connective tissue destruction and bone resorption. In contrast, cariogenic biofilms are primarily composed of acidogenic and aciduric species, notably *S. mutans*, which ferment dietary carbohydrates to produce organic acids that demineralize enamel. These distinct etiologies, microbial compositions, and tissue targets mean that findings from caries models cannot be directly extrapolated to periodontitis. Similarly, monospecies infection models, while useful for mechanistic studies, do not fully recapitulate the polymicrobial interactions and spatial organization of subgingival periodontal biofilms, and evidence derived from such models should be interpreted as indirect mechanistic insight.

### 2.2 Biofilm-mediated pathogenicity

The pathogenic potential of periodontal biofilms extends beyond the direct effects of bacterial virulence factors. The EPS matrix impedes the penetration of antibiotics, antimicrobial peptides, and immune cells, reducing their access to biofilm-embedded bacteria (Pinto et al., 2020; Lv et al., 2023). Bacteria within biofilms exhibit metabolic heterogeneity, with deep-layer cells entering a low-metabolic or dormant state, and this phenotypic adaptation significantly reduces their susceptibility to antibiotics that target active metabolic processes (Stewart, 2002; Mishra et al., 2023). Biofilm formation promotes bacterial cooperation and virulence factor production through quorum sensing, a cell-to-cell communication mechanism that coordinates gene expression within the biofilm community (Zhao et al., 2025a; Shan, et al., 2025; Zhao et al., 2024). Furthermore, biofilms can subvert host immune responses, promoting a chronic inflammatory state that perpetuates tissue destruction rather than resolving infection (Hajishengallis, 2015; Kinane et al., 2017).

### 2.3 Limitations of current therapeutic approaches

Current periodontal therapy relies primarily on mechanical debridement combined with adjunctive antimicrobial agents (Kinane et al., 2017). However, mechanical debridement cannot completely eliminate biofilms from inaccessible areas such as deep periodontal pockets, furcations, and root surface irregularities. Prolonged or repeated antibiotic use promotes the emergence of resistant bacterial strains, while the EPS matrix and metabolic dormancy of biofilm-embedded bacteria render them highly tolerant to antibiotics, often requiring concentrations far exceeding those achievable in clinical settings (Stewart, 2002; Fulaz et al., 2019). Additionally, persistent inflammation driven by residual biofilm creates a self-perpetuating cycle of tissue destruction that impairs healing and regeneration (Hajishengallis, 2015). These limitations underscore the urgent need for novel therapeutic strategies that can effectively disrupt biofilms, overcome resistance mechanisms, and promote tissue regeneration (Mishra et al., 2023; Choi et al., 2023).

## 3 Nanozyme-mediated antibiofilm mechanisms

### 3.1 ROS-mediated bacterial killing

The primary antimicrobial mechanism of nanozymes involves the catalytic generation of ROS. Nanozymes with peroxidase-like activity catalyze the conversion of H_2_O_2_ into hydroxyl radicals (·OH) in acidic microenvironments, while nanozymes with oxidase-like activity can utilize O_2_ directly to generate superoxide anions (O_2_·^-^) without exogenous H_2_O_2_ (Gao et al., 2007; Gao et al., 2021; Alfei and Catenaro, 2024). These ROS species exert broad-spectrum antimicrobial effects through multiple mechanisms, including oxidation of membrane lipids leading to increased permeability and cell lysis, modification and inactivation of essential bacterial proteins, and induction of strand breaks and base modifications in bacterial DNA (Chen et al., 2018; Song et al., 2024). Unlike conventional antibiotics that rely on single molecular targets, ROS-based antimicrobial mechanisms are difficult for bacteria to evade through genetic mutation or metabolic reprogramming, making this resistance-independent mechanism particularly valuable for combating biofilm-associated infections where antibiotic tolerance is a major challenge (Zhou and Wang, 2022; Wang et al., 2022a; Wang et al., 2022b).

The catalytic efficiency of nanozymes can be substantially enhanced through rational design and environmental stimuli. For example, the acidic microenvironment characteristic of inflamed periodontal pockets (pH 5.5–6.5) favorably activates peroxidase-like nanozymes, which exhibit optimal activity under mildly acidic conditions (Gao et al., 2007; Shen et al., 2023). Moreover, photothermal and photodynamic strategies can be integrated with nanozyme platforms to achieve synergistic ROS amplification, whereby near-infrared irradiation elevates local temperature to accelerate catalytic turnover (Li Z. et al., 2026; Pang et al., 2020). Single-atom nanozymes, in which individual metal atoms are dispersed on supports such as carbon nitride or metal-organic frameworks, represent the state of the art in maximizing atom utilization and catalytic specificity (Jin et al., 2023; Zhu and Liu, 2022). These advancements have enabled nanozymes to achieve catalytic efficiencies approaching those of natural enzymes, while maintaining the robustness and tunability inherent to nanomaterial platforms.

### 3.2 EPS matrix degradation

Nanozymes can directly degrade the EPS matrix through catalytic and oxidative mechanisms. The EPS matrix is composed of polysaccharides, proteins, and eDNA, each of which can be targeted by specific enzymatic activities (Flemming and Wingender, 2010; Pinto et al., 2020). Nanozymes with glycoside hydrolase-like activities can hydrolyze the structural polysaccharides within the EPS matrix, while nanozymes with protease-like activities can degrade the protein components (Li et al., 2022; Sheng and Zhang, 2023). Additionally, nanozymes with nuclease-like activities can cleave extracellular DNA, which serves as a critical structural scaffold within many biofilms (Zou et al., 2025; Pan et al., 2022). By degrading the EPS matrix, nanozymes disrupt the physical barrier that protects biofilm-embedded bacteria, rendering them more susceptible to antimicrobial agents and host immune defenses (Lv et al., 2023; Fulaz et al., 2019). This dual-action strategy, simultaneously killing bacteria and dismantling the protective matrix, represents a key advantage of nanozyme-based antibiofilm therapy.

### 3.3 Disruption of bacterial adhesion and quorum sensing

Nanozymes can interfere with early stages of biofilm formation by inhibiting bacterial adhesion to tooth surfaces. Surface modifications of nanozymes can create anti-adhesive properties that prevent initial bacterial colonization (Benoit et al., 2019). Inspired by quorum-quenching strategies, certain nanozyme platforms may potentially interfere with bacterial quorum sensing. Although direct nanozyme-mediated quorum sensing inhibition has not been extensively demonstrated, studies using quorum-quenching agents such as coumarin and the lactonase Est816 have established the concept that disrupting bacterial communication can attenuate biofilm development and reduce virulence factor production (Zhao et al., 2025b; Guo, et al., 2025; He et al., 2022). These observations suggest that nanozymes with appropriate surface modifications could be designed to incorporate quorum-quenching functions, though this remains an area requiring further investigation.

### 3.4 Modulation of the inflammatory microenvironment

Beyond direct antimicrobial effects, nanozymes can modulate the inflammatory microenvironment that characterizes periodontitis. Nanozymes with catalase- and superoxide dismutase (SOD)-like activities can scavenge excessive ROS at inflammatory sites, reducing oxidative tissue damage (Liu et al., 2023; Kurian et al., 2024). This dual functionality, combining antimicrobial and antioxidant activities, is particularly valuable for periodontitis, where the disease process involves both persistent infection and chronic inflammation (Hajishengallis, 2015; Luo et al., 2025). By reducing ROS levels, antioxidant nanozymes such as CeO_2_ and MnO_2_-based systems can alleviate oxidative stress and tissue damage, promote macrophage M2 polarization toward a pro-resolving phenotype, and create a favorable microenvironment for tissue repair and bone regeneration. This immunomodulatory capacity, which links direct ROS scavenging to downstream osteogenic effects, distinguishes nanozymes from conventional antimicrobials that address only the infectious component of the disease (Xu et al., 2023; Hu et al., 2023; Kurian et al., 2024) (Figure 1).

**FIGURE 1:**
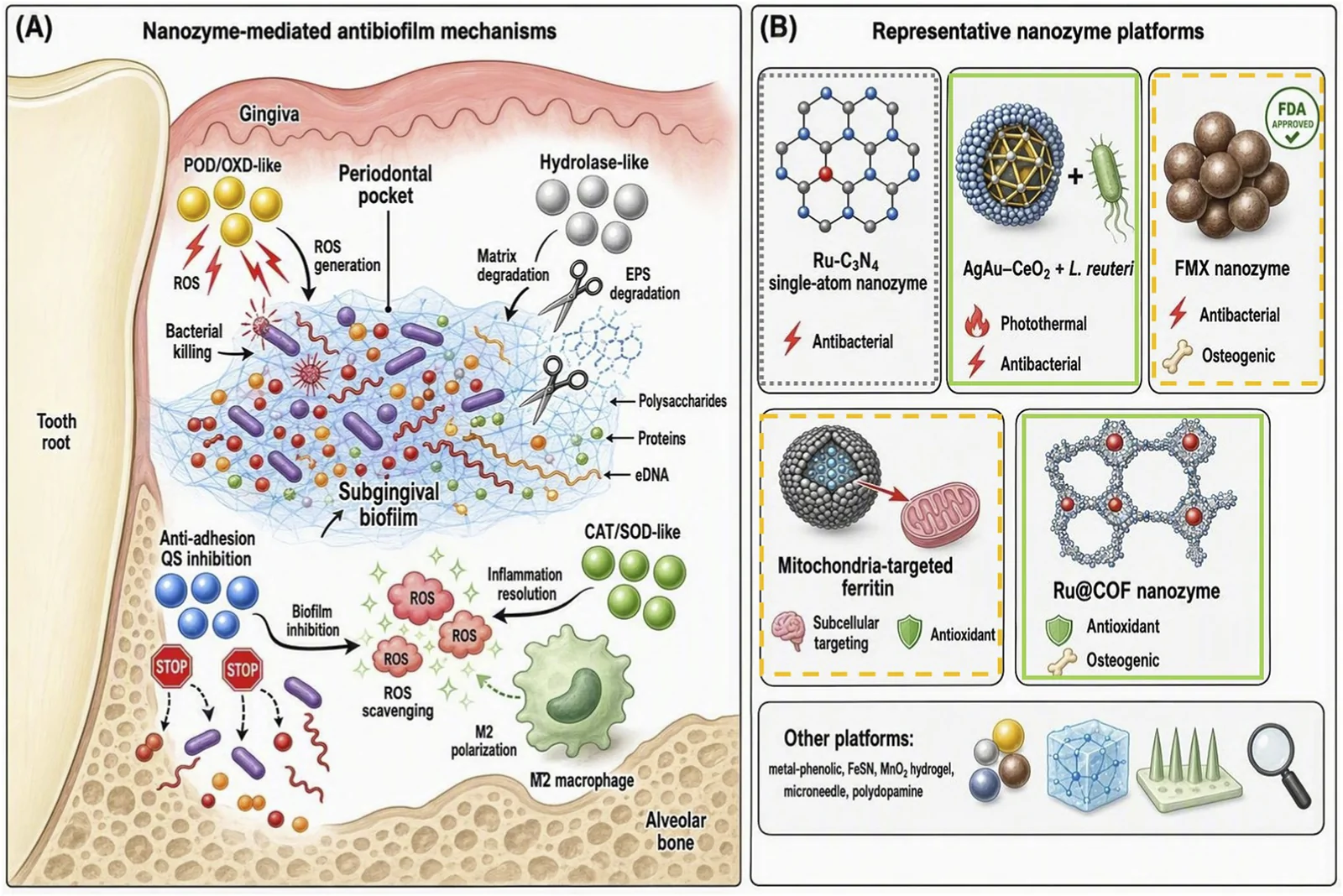
Schematic illustration of nanozyme-mediated antibiofilm mechanisms in periodontitis and representative nanozyme platforms for periodontal therapy. **(A)** Nanozymes combat periodontal biofilms through four complementary mechanisms: ROS generation via POD/OXD-like activities, EPS degradation via hydrolase-like activities, inhibition of bacterial adhesion and quorum sensing, and ROS scavenging via CAT/SOD-like activities to modulate inflammation. **(B)** Representative nanozyme platforms evaluated in preclinical models with translational potential for periodontal therapy include Ru-C_3_N_4_ single-atom nanozymes, AgAu-CeO_2_ heterojunction nanozymes with probiotics, ferumoxytol (FMX) nanozymes, mitochondria-targeted ferritin nanozymes, and Ru@COF nanozymes. Legend: Solid lines/green elements indicate direct periodontal evidence; dashed lines/orange elements indicate indirect evidence (apical periodontitis or other oral diseases); dotted lines/gray elements indicate hypothetical mechanisms or non-oral biofilm evidence.

### 3.5 The ROS paradox: balancing antimicrobial and antioxidant activities

A fundamental challenge in nanozyme-based periodontal therapy lies in the apparent paradox between ROS-generating and ROS-scavenging activities. Peroxidase- and oxidase-like nanozymes generate bactericidal ROS (·OH, O_2_·^-^) to kill biofilm-embedded bacteria, whereas catalase- and SOD-like systems remove ROS to limit tissue injury. Understanding how these opposing functions are controlled is critical for therapeutic application. pH-dependent activity provides a key mechanism of spatial control. Peroxidase-like nanozymes exhibit optimal activity under acidic conditions (pH 5.5–6.5), which characterize inflamed periodontal pockets and biofilm microenvironments (Gao et al., 2007). In contrast, catalase- and SOD-like nanozymes typically function more effectively at neutral pH (7.0–7.4), which predominates in healthy tissues. This pH dependency allows nanozymes to preferentially generate ROS at infection sites while limiting oxidative damage to surrounding healthy tissues.

Concentration-dependent effects further modulate ROS activity. At low concentrations, ROS function as signaling molecules that activate host defense pathways. At high concentrations, ROS induce oxidative damage. Nanozyme design can exploit this threshold effect by tuning catalytic activity to achieve bactericidal concentrations at the biofilm interface while remaining below cytotoxic thresholds for host cells. Spatial control can be achieved through subcellular targeting. For example, mitochondria-targeted ferritin nanozymes deliver SOD/CAT activity directly to mitochondria, the primary source of intracellular ROS, enabling efficient ROS scavenging precisely where it is needed to inhibit pyroptosis (Ma et al., 2025). Temporal control is afforded by stimuli-responsive delivery systems. ROS-responsive or pH-responsive nanozyme formulations release or activate their catalytic functions only upon exposure to the infected microenvironment, minimizing systemic exposure. Finally, differential susceptibility between bacterial and mammalian cells contributes to therapeutic selectivity. Bacterial cells lack the robust antioxidant defense systems (e.g., glutathione, thioredoxin, catalase) present in mammalian cells, making them more vulnerable to ROS-mediated damage. Host cells also possess superior DNA repair mechanisms, further increasing their tolerance to oxidative stress. These factors collectively enable nanozymes to achieve antibacterial activity without significant damage to periodontal host cells, though careful optimization of dose and delivery is required for each platform.

## 4 Representative nanozyme platforms for periodontitis

### 4.1 Ru-C_3_N_4_ single-atom nanozymes

Single-atom nanozymes represent the frontier of nanozyme engineering, where metal atoms are atomically dispersed on a support to maximize catalytic efficiency (Jin et al., 2023; Hua and Yang, 2024). This platform was selected to exemplify atom-economical design, as single-atom dispersion maximizes metal utilization and provides uniform active sites for enhanced peroxidase-like activity. Ruthenium single-atom nanozymes (Ru-C_3_N_4_) have demonstrated potent peroxidase-like activity comparable to that of natural horseradish peroxidase (Wang et al., 2024). It should be noted that this platform was originally studied in an otitis media mouse model and against *Staphylococcus aureus* biofilms, rather than in a periodontal model. Nevertheless, these nanozymes have shown efficacy against biofilm-associated infections through robust ROS generation, achieving effective biofilm degradation and bacterial elimination at low concentrations. Their potent antibiofilm activity suggests translational potential that warrants investigation in periodontal applications. The single-atom design maximizes atom utilization efficiency and provides uniform active sites, offering advantages in catalytic activity and selectivity over conventional nanoparticle-based nanozymes (Jin et al., 2023; Zhu and Liu, 2022). A separate study on a copper-based single-atom system demonstrated that spatiotemporally guided delivery of single-atom bionanozymes can improve targeting specificity for antibiofilm treatment, reducing off-target effects and enhancing therapeutic efficacy (Huang et al., 2024). While this platform differs from Ru-C_3_N_4_ in composition, its design principles may inform the development of ruthenium-based systems for periodontal applications.

### 4.2 AgAu–CeO_2_ heterojunction nanozymes integrated with probiotics

A notable example of advanced nanozyme design is the AgAu–CeO_2_. The primary study of this platform was reported by Li M and colleagues, who developed the AgAu–CeO_2_ heterojunction integrated with *Lactobacillus reuteri* and validated its efficacy in a rat periodontitis model (Li M. et al., 2026). This system illustrates multi-mechanism synergy, where heterojunction engineering, photothermal activation, and probiotic-mediated pH modulation are combined to overcome the limited catalytic efficiency of single-component nanozymes. This system employs a triple-modulation strategy to enhance catalytic activity: photothermal heating by near-infrared light irradiation that induces localized surface plasmon resonance and elevates local temperature to enhance catalytic efficiency; interfacial electron transfer through the AgAu–CeO_2_ heterojunction that promotes Ce^3+^/Ce^4+^ redox cycling to sustain catalytic activity; and pH modulation as *L. reuteri* secretes lactic and acetic acids to create an acidic microenvironment favorable for peroxidase-like activity. This triple-modulation strategy markedly enhances the POD-like activity of CeO_2_, triggering a robust ROS burst that effectively disrupts periodontal biofilms and eliminates pathogenic bacteria while preserving beneficial commensal populations. The integration of probiotics with nanozymes represents a synergistic approach that combines catalytic antibiofilm activity with microbiome rebalancing.

### 4.3 Ferumoxytol (FMX) nanozymes

Ferumoxytol is an FDA-approved formulation of iron oxide nanoparticles originally developed for iron replacement therapy (Vasanawala and Cohen, 2016; Huang et al., 2022). Its selection highlights the value of drug repurposing leveraging an existing clinically approved nanomaterial for catalytic antibiofilm therapy, thereby bypassing some regulatory and safety hurdles associated with *de novo* nanozyme development. Recent research has demonstrated its potential as a nanozyme for biofilm infections via catalytic activation of hydrogen peroxide (Babeer et al., 2024). In a clinical study of apical periodontitis as an endodontic infection distinct from marginal periodontitis in terms of location, microbiology, and standard treatment, a single application of FMX with H_2_O_2_ achieved robust antibiofilm activity comparable to sodium hypochlorite, the current gold standard, without adverse effects. Notably, FMX binds efficiently to bacterial pathogens and remains catalytically active without being affected by dental tissue components. While this evidence is derived from apical periodontitis and thus constitutes indirect evidence for marginal periodontitis, it demonstrates the translational potential of nanozyme-based catalytic antibiofilm therapy in oral infections and warrants investigation in marginal periodontitis models. FMX activates stem cell pluripotency and WNT/NOTCH signaling that induces osteogenic capacity, demonstrating that FMX nanozymes offer a regenerative approach to antimicrobial therapy with unique stem cell-stimulatory properties.

### 4.4 Mitochondria-targeted ferritin nanozymes

A mitochondria-targeted ferritin nanozyme has been engineered to modulate the ROS-pyroptosis axis in periodontitis (Ma et al., 2025). This platform was chosen to demonstrate subcellular targeting precision, as mitochondria are the primary source of intracellular ROS, and delivering nanozyme activity directly to this organelle enables more efficient ROS scavenging and pyroptosis inhibition. This platform achieves lysosomal escape and mitochondrial delivery through rational integration of genetic engineering and protein expression techniques. The MnO_2_ nanozymes bioengineered within ferritin nanocages display SOD/CAT mimicry, scavenging mitochondrial ROS, restoring mitochondrial homeostasis, and inhibiting caspase-1-dependent pyroptosis. In periodontitis mice, the platform demonstrated 24-h retention, attenuated alveolar bone loss, and reduced pyroptosis markers. This study pioneers a mitochondria-targeting ferritin nanozyme that coordinately regulates the ROS-pyroptosis axis, providing a promising therapeutic strategy for periodontitis by simultaneously addressing oxidative stress and inflammatory cell death (Kurian et al., 2024; Li M. et al., 2025).

### 4.5 Ru@COF nanozymes

Ruthenium-coordinated covalent organic framework (Ru@COF) nanozymes have been developed to simultaneously scavenge ROS and promote osteogenesis in periodontitis (Qin et al., 2025). This platform exemplifies dual-function integration, combining antioxidant activity with osteogenic promotion within a single covalent organic framework, thereby addressing both inflammatory and regenerative dimensions of periodontal therapy. These nanozymes exhibit SOD/CAT-like activities that reduce oxidative stress at inflammatory sites, while their porous COF structure enables efficient drug loading and controlled release. By lowering intracellular ROS levels, these nanozymes alleviate oxidative damage to osteoblast precursors and facilitate the activation of the BMP-2/Runx2 signaling pathway, which is essential for osteoblast differentiation and bone formation. In ligature-induced periodontitis models, Ru@COF nanozymes significantly reduced osteoclast counts, preserved alveolar bone, and promoted bone regeneration through activation of the BMP-2/Runx2 signaling pathway. The combination of antioxidant and osteogenic activities in a single platform address both the inflammatory and regenerative dimensions of periodontal therapy, representing a multifunctional approach to periodontal tissue engineering.

### 4.6 Other notable nanozyme platforms

In a study directly investigating periodontitis, Li and colleagues developed a multifunctional yolk-shell nanozyme (Au@CeO_2_-DMF) that integrates gold and cerium oxide into a photothermally regulated high-efficiency nanozyme platform loaded with dimethyl fumarate (Li T. et al., 2025). This system comprehensively addresses oxidative stress-induced pathophysiological processes through three complementary mechanisms: antioxidant activity, mitochondrial maintenance, and immune modulation. In a rat periodontitis model, local Au@CeO_2_-DMF photothermal therapy effectively alleviated ROS-induced tissue damage and restored periodontal homeostasis. This study demonstrates that CeO_2_-based nanozymes can achieve long-term therapeutic effects by simultaneously targeting multiple pathological processes. Other CeO_2_-based platforms have also been proposed as strategies to overcome biofilm resistance and inflammatory dysregulation in periodontitis (Hosseini Hooshiar et al., 2024; Liu et al., 2024). In a periodontitis model, ultrasound-driven piezoelectric-nanozyme micromotors (BaTiO_3_@CeO_2_-Pt) were developed to dynamically regulate ROS generation and clearance, addressing bacterial infection, inflammation, and bone regeneration simultaneously (Chen et al., 2025). Microenvironment-responsive metal-phenolic nanozyme release platforms have been evaluated in periodontitis models, demonstrating antibacterial, ROS scavenging, and osteogenesis capabilities (Xu et al., 2023; Li D. et al., 2025). In a periodontitis model, polydopamine dual-modal nanotherapy synergized photothermal antibacterial and nanozyme anti-inflammatory effects (Guo et al., 2025). Dual nanozyme-loaded core-shell microneedle patches with antibacterial and neutrophil extracellular trap (NET)-degrading properties have been developed for periodontitis treatment (Shan et al., 2025). This platform represents a direct periodontal application and demonstrates the potential of transdermal microneedle-based delivery for localized periodontal therapy. In a periodontitis model, histidine-doped FeSN nanozymes with ultra-high peroxidase-like activity were applied for both detection and treatment of biofilm-induced periodontitis (Shen et al., 2023). Catechol-modified MnO_2_-nanozyme-reinforced hydrogels have been evaluated in periodontitis models, demonstrating improved antioxidant and antibacterial capacity (Hu et al., 2023).

The iron oxide nanozyme platform has also been adapted for caries prevention by stabilizing stannous fluoride, enabling targeted biofilm killing through combined catalytic and antimicrobial actions (Huang et al., 2023). Although this application addresses dental caries rather than periodontitis, it demonstrates the versatility of nanozyme platforms as carriers and catalytic enhancers when combined with existing therapeutic agents. Furthermore, Complexed micelle systems based on CeO_2_ nanozymes have been investigated in periodontitis models for concurrent bone remodeling and ROS scavenging (Xu et al., 2026). These diverse platforms collectively demonstrate that nanozyme engineering can be tailored to address the specific pathological features of periodontitis, from biofilm disruption to immune modulation and bone regeneration, offering a versatile toolkit for precision periodontal therapy (Table 1).

**TABLE 1:** Representative nanozyme platforms for periodontitis.

Evidence level	Nanozyme platform	Composition	Enzyme-mimetic activities	Mechanisms of action	Experimental model and microbial system	Dose and treatment duration	Key limitations and translational status	References
Direct periodontal evidence	AgAu–CeO_2_ heterojunction + probiotics	AgAu nanocages + CeO_2_ + *L. reuteri*	POD-like (triple-enhanced)	Photothermal heating, electron transfer, pH modulation	Rat periodontitis model; *P. gingivalis* and polymicrobial biofilms	Not specified	Requires NIR irradiation. Biocompatibility reported but long-term safety unknown	Li M. et al. (2026)
​	Mitochondria-targeted ferritin nanozyme	Ferritin nanocages with MnO_2_	SOD/CAT-like	mtROS scavenging, pyroptosis inhibition	Murine periodontitis model	Not specified	24 h retention. Evidence from conference proceeding; requires full-length validation	Ma et al. (2025) [conference abstract]
​	Ru@COF nanozymes	Ru-coordinated covalent organic framework	SOD/CAT-like	ROS scavenging, BMP-2/Runx2 activation	Ligature-induced periodontitis model; osteoblast precursors	Not specified	Dual antioxidant and osteogenic functions. COF biodegradability requires investigation	Qin et al. (2025)
​	Au@CeO_2_ yolk-shell nanozyme	Au@CeO_2_ yolk-shell + DMF	SOD/CAT-like (antioxidant)	Antioxidant, mitochondrial maintenance, immune modulation, photothermal therapy	Rat periodontitis model	Not specified	Requires NIR irradiation (635 nm). DMF activates NRF2 pathway, inhibits NLRP3 inflammasome. Multifunctional platform	Li T. et al. (2025)
​	Piezoelectric-nanozyme micromotors	BaTiO_3_@CeO_2_-Pt	SOD/CAT/POD-like	ROS generation/clearance, dynamic regulation	Periodontitis models	Not specified	Requires ultrasound activation. Delivery complexity may limit translation	Chen et al. (2025)
​	Metal-phenolic nanozyme	Metal-phenolic network	Antibacterial/antioxidant	On-demand release, ROS scavenging, osteogenesis	Periodontitis models	Not specified	Microenvironment-responsive. Metal ion release and long-term safety require assessment	Xu et al. (2023); Li D. et al. (2025)
​	Histidine-doped FeSN nanozyme	FeSN with histidine doping	POD-like (ultra-high)	ROS generation, biofilm detection and treatment	Periodontitis models	Not specified	Dual detection/treatment function. Fe/S/N biodegradability favorable	Shen et al. (2023)
​	Catechol-modified MnO_2_ hydrogel	MnO_2_ nanozyme in catechol hydrogel	SOD/CAT-like	Antioxidant, antibacterial, sustained release	Periodontitis models	Not specified	Improved antioxidant and antibacterial capacity. Hydrogel retention in pockets requires validation	Hu et al. (2023)
​	Polydopamine dual-modal nanotherapy	Polydopamine-based nanocomposite	POD-like + anti-inflammatory	Photothermal antibacterial, ROS scavenging	Periodontitis models	Not specified	Requires NIR irradiation. Synergistic effects demonstrated	Guo et al. (2025)
​	Dual nanozyme microneedle patch	Dual nanozymes in core-shell microneedle	Antibacterial + NETs-degradation	Antibacterial, NETs clearance, anti-inflammatory	Periodontitis model	Not specified	Direct periodontal application. Transdermal delivery promising. Requires further validation	Shan et al. (2025)
Indirect evidence	Ru-C_3_N_4_ single-atom nanozymes	Ru atoms on g-C_3_N_4_ support	POD-like	ROS generation, biofilm degradation	*S. aureus* biofilm; otitis media mouse model (not periodontal)	0.06 mg mL^−1^; single dose	Potent activity but requires validation in periodontal models. Low metal load suggests favorable safety profile	Wang et al. (2024)
​	Ferumoxytol (FMX) nanozymes	Iron oxide nanoparticles (FDA-approved)	POD-like	Catalytic H_2_O_2_ activation, free radical generation	Apical periodontitis clinical study; polymicrobial biofilms	Single application with H_2_O_2_	Clinically tested in different disease context. Requires exogenous H_2_O_2_. Promotes stem cell growth via WNT/NOTCH activation	Babeer et al. (2024)

Abbreviations: POD, peroxidase; SOD, superoxide dismutase; CAT, catalase; mtROS, mitochondrial reactive oxygen species; NETs, neutrophil extracellular traps; COF, covalent organic framework; NIR, near-infrared; DMF, dimethyl fumarate.

### 4.7 Critical appraisal of nanozyme platforms

While the platforms discussed above demonstrate promising antibiofilm activity, several critical considerations must be addressed to assess their translational potential. For metal-based nanozymes (including Ru, Au, Ag, Ce, Fe, and Cu-based systems), the release of metal ions raises concerns regarding local tissue toxicity, systemic biodistribution, and long-term accumulation. Studies investigating the gingival tissue penetration and clearance of these materials remain limited. Genotoxicity assessment is similarly under-reported, particularly for systems that generate high levels of ROS, which may inadvertently damage host cell DNA. Immunogenicity represents another knowledge gap, as the potential for nanozyme-induced complement activation or cytokine release has not been systematically evaluated for periodontal applications. The impact on the oral microbiome is also poorly characterized; while nanozymes are designed to target pathogenic biofilms, their broad-spectrum ROS generation may disrupt commensal microbial communities. Biodegradation and clearance pathways vary substantially across platforms: iron oxide-based systems (e.g., FMX) may be cleared via renal or hepatic routes, while non-biodegradable materials (e.g., Au, COF-based systems) raise concerns regarding chronic retention. Furthermore, most existing studies have utilized monospecies or simplified polymicrobial models, and efficacy data in clinically relevant subgingival biofilm models are scarce.

## 5 Advanced delivery strategies for periodontal nanozyme therapy

For periodontal applications, delivery systems must contend with several unique anatomical and physiological features. Periodontal pockets vary in depth (3–12 mm) and morphology, with narrow entrances, irregular root surface contours, and furcation involvements that limit access for topically applied therapeutics (Choi et al., 2023). The continuous flow of gingival crevicular fluid (GCF) approximately 0.5–2 μL per minute per pocket creates a washout effect that dilutes and clears locally applied agents within minutes to hours, necessitating formulations with sustained retention or mucoadhesive properties. Mucoadhesion, mediated by electrostatic interactions, hydrogen bonding, or hydrophobic interactions, is therefore essential for prolonged residence time. Local retention can be enhanced through injectable hydrogels, which exhibit shear-thinning properties for easy administration and rapid recovery of gel structure upon injection (Hu et al., 2023; Xiao et al., 2025). Once in place, the hydrogel matrix provides sustained retention at the diseased site, resisting displacement by gingival crevicular fluid flow and allowing prolonged, localized release of nanozymes to disrupt biofilms and modulate inflammation. The continuous flow of gingival crevicular fluid can dilute and wash away locally applied agents, while the EPS matrix within biofilms impedes the penetration of nanoparticles (Fulaz et al., 2019; Lv et al., 2023). Local delivery systems offer advantages over systemic administration for periodontal therapy, including higher local drug concentrations and reduced systemic side effects (Choi et al., 2023; Benoit et al., 2019). Various delivery systems have been developed for nanozyme delivery, including injectable hydrogels that can be placed directly into periodontal pockets to provide sustained release of nanozymes, nanoparticle carriers in which nanozymes can be encapsulated or conjugated for enhanced stability and targeted delivery, and stimuli-responsive systems that release nanozymes in response to specific triggers such as acidic pH, elevated ROS, or bacterial enzymes (Zou et al., 2025; Wang et al., 2022b; Lin, et al., 2022; Li D. et al., 2025). Microneedle-based transdermal delivery has emerged as a promising approach for periodontal applications, enabling minimally invasive penetration of the gingival tissue for targeted nanozyme delivery (Shan et al., 2025; Zhang et al., 2025). Penetration into polymicrobial subgingival biofilms requires nanoscale dimensions (typically <100 nm) and surface properties that facilitate diffusion through the EPS matrix; surface modifications such as PEGylation or charge optimization can enhance penetration (Somasundar et al., 2023; Sheng and Zhang, 2023). Finally, delivery systems should be compatible with scaling and root planing (SRP), the standard mechanical debridement procedure. Ideally, nanozyme formulations could be applied as adjuncts to SRP, with delivery systems that remain effective despite disruption of the pocket environment.

Emerging delivery paradigms include nanozyme-functionalized micromotors that achieve active propulsion through catalytic bubble generation, enabling deep penetration into biofilm matrices that passive diffusion cannot achieve (Tran et al., 2024; Huang et al., 2022). Magnetic-field-guided nanozyme swarms have demonstrated precise spatial targeting and collective catalytic activity, offering controllable biofilm elimination (Huang et al., 2022). Additionally, biomimetic approaches using cell membrane-coated nanozymes, such as erythrocyte or macrophage membrane camouflaging, have improved immune evasion and prolonged circulation time while retaining catalytic activity (Hu et al., 2023). These advanced delivery strategies represent critical enablers for translating nanozyme catalytic potential into effective *in vivo* periodontal therapy.

## 6 Challenges and future perspectives

Despite encouraging preclinical evidence, the biosafety of nanozymes for periodontal therapy remains a significant concern. The long-term accumulation of metal active centers (Fe, Cu, Mn, Ce, Ru, etc.) in tissues remains unclear (Hasan et al., 2025; Zandieh and Liu, 2021). Catalytically produced ROS, while killing pathogens, may cause oxidative DNA damage in host cells (Alfei and Catenaro, 2024; Liu et al., 2023). The risk of abnormal immune activation or suppression by nanomaterials requires systematic evaluation, and existing safety data are largely derived from short-term animal studies, with long-term clearance and chronic toxicity studies being almost absent (Ma, 2024; Hetta et al., 2023).

Several translational barriers impede the clinical development of nanozyme-based periodontal therapies. Significant discrepancies exist between *in vitro* potency and *in vivo* efficacy, as the complex periodontal microenvironment substantially reduces actual catalytic efficiency (Hosseini Hooshiar et al., 2024; Li M. et al., 2025). Existing rodent models cannot fully recapitulate the pathological features of human periodontitis, and batch-to-batch consistency of nanozyme size, morphology, and surface chemistry is difficult to guarantee in scaled production (Chen et al., 2025; Luo et al., 2025). As a novel therapeutic entity, the regulatory classification and approval pathway for nanozymes currently lacks a clear regulatory framework (Ma, 2024). Future research should prioritize the development of standardized protocols for toxicity evaluation, including long-term biocompatibility, genotoxicity, and immunogenicity assessment. The design of biodegradable and renally clearable nanozymes is needed to avoid long-term bioaccumulation (Meng et al., 2026; Li M. et al., 2025). Artificial intelligence (AI)-assisted nanozyme design holds particular promise for accelerating catalyst discovery. For instance, machine learning models have been trained on compositional and catalytic data to predict peroxidase-like activity, enabling high-throughput screening of potential nanozyme candidates before experimental validation (Ge et al., 2025). Progression from preclinical studies to first-in-human trials with appropriate outcome measures for periodontal indications is essential. Exploration of synergistic combinations of nanozymes with antibiotics, photothermal therapy, or other modalities may enhance therapeutic efficacy (Pang et al., 2020; Youf and Seguin, 2021). Development of patient-specific nanozyme formulations based on disease severity, pathogen profile, and host immune status represents a promising direction for personalized periodontal therapy (Luo et al., 2025; Zhao et al., 2026).

Looking ahead, artificial intelligence-assisted nanozyme design holds transformative potential. Machine learning models can predict optimal compositions, surface modifications, and catalytic active sites by mining structure–activity relationships across large nanozyme datasets, dramatically accelerating the discovery pipeline (Li et al., 2022; Huang et al., 2024). Combinatorial strategies that pair nanozymes with antimicrobial photodynamic therapy, sonodynamic therapy, or immunomodulatory agents may achieve synergistic effects at lower individual doses, reducing potential toxicity (Pang et al., 2020; Youf and Seguin, 2021; Songca and Oluwafemi, 2022). Furthermore, the development of nanozyme-functionalized dental materials such as adhesive resins, bone graft substitutes, and guided tissue regeneration membranes, could provide sustained antibiofilm protection at the surgical site following periodontal procedures (Zhao et al., 2026; Chen et al., 2021). Ultimately, the successful clinical translation of nanozyme-based periodontal therapy will require interdisciplinary collaboration among materials scientists, periodontitis researchers, regulatory agencies, and clinical investigators, guided by rigorous safety standards and well-designed clinical trials.

## 7 Conclusion

Nanozymes offer a multifunctional platform against periodontal biofilms through ROS-mediated killing, EPS degradation, quorum sensing disruption, and inflammation modulation. Multiple preclinical platforms have demonstrated efficacy in biofilm eradication, inflammation reduction, and bone regeneration. Despite these advances, significant challenges in long-term biosafety, metal-ion accumulation, and clinical translation persist. Future research should prioritize standardized safety evaluation, biodegradable nanozyme designs, and rigorous clinical validation. The convergence with single-atom engineering, AI-assisted design, and smart responsive delivery systems has the potential to accelerate the development of next-generation antibiofilm therapeutics for periodontitis. However, realizing this potential will require addressing the significant biosafety, manufacturing, and regulatory challenges discussed above.
